# Response to the COVID-19 pandemic among the Ukrainian students: Coping strategies and psychological adjustment

**DOI:** 10.1192/j.eurpsy.2023.1707

**Published:** 2023-07-19

**Authors:** T. Vasheka, S. Tukaiev, O. Dolgova

**Affiliations:** 1 Faculty of Linguistics and Social Communications, Aviation psychology department, National Aviation University, Kyiv, Ukraine; 2 Faculty of Communication, Culture, and Society, Institute of Public Health, Università della Svizzera italiana, Lugano, Switzerland; 3 Faculty of Linguistics and Social Communications, Aviation psychology department, Università della Svizzera italiana, Kyiv, Ukraine

## Abstract

**Introduction:**

The COVID-19 pandemic has brought significant transformations in the social life due to the isolation itself and the effect of quarantine restrictions, which together affect the psychological health and well-being in different countries.

**Objectives:**

The aim of this study was to establish the emotional and behavioral students’ responses to the coronavirus pandemic, to assess the dominant coping strategies and the prevalence of neurotic states and stress level among students.

**Methods:**

For this study, a specially designed questionnaire with a set of psychodiagnostic methods was used to diagnose the manifestations of neurotic conditions in students (Clinical questionnaire for the identification and evaluation of neurotic conditions by K.K. Yakhin, D.M. Mendelevich), the level of psychological stress and the main coping strategies (questionnaire " Coping strategies” by R. Lazarus). The sample consisted of 213 respondents, students of Kyiv Universities (119 female)

**Results:**

Among Ukrainian students, the dominant reactions to the COVID-19 pandemic are depressive disorders, anxiety and fears, a third of the students self-reported autonomic disorders. The stress level is moderate. Female reacted to the pandemic situation with more serious mental health disorders compared to male. The use of all coping strategies was recorded at a high level of tension, which indicates that students are already exhausting their adaptive potential and are actually maladapted in the current conditions.

**Conclusions:**

The study confirms the negative impact of the COVID-19 pandemic on the mental health of young people. The available mental resources to overcome a difficult situation are exhausted, so students prefer to avoid and ignore stressful information. This gives rise to the need for psychological support and educational activities on health techniques.

**Disclosure of Interest:**

None Declared

